# Etiology, clinical manifestations, and management methods of cryptitis beside the preputial frenulum in men

**DOI:** 10.1186/s12610-024-00219-0

**Published:** 2024-01-16

**Authors:** Wenge Fan, Qingsong Zhang, Zhijiang Fan, Mei Wei, Yuan Zhu

**Affiliations:** 1 Department of Dermatology, First People’s Hospital of Changshu City, Changshu Hospital Affiliated to Soochow University, Jiangsu Province, 1 Shuyuan Street, Changshu City, 215500 P. R. China; 2 Department of Dermatology, Nantong University Affiliated Qidong Hospital, Qidong, Jiangsu Province 226200 P. R. China; 3 Department of Urinary Surgery, First People’s Hospital of Changshu City, Changshu Hospital Affiliated to Soochow University, Changshu, Jiangsu Province 215500 P. R. China

**Keywords:** Cryptitis, Preputial frenulum, Men, Cryptite, Frein du prepuce, Hommes

## Abstract

**Background:**

Inflammatory diseases may occur within the crypt beside the preputial frenulum in men. This study was performed to gain an understanding of the etiology, clinical manifestations, and management methods of cryptitis beside the preputial frenulum in men.

**Results:**

Thirteen patients treated for cryptitis beside the preputial frenulum served as the observation group, and 40 healthy individuals served as the control group. The patients’ clinical manifestation was the presence of a yellowish oily substance embedded in the crypt. Wiping off the substance revealed a conical blind cavity-like structure with an opening diameter of 1 to 5 mm (2.8 ± 1.3 mm) and depth of 1 to 4 mm (2.5 ± 1.1 mm). No blind cavity-like structures in the crypt were found in the control group. The shortest distance between the opening edges of the bilateral crypts in the observation and control groups was 6 to 14 mm (10.3 ± 2.4 mm) and 2 to 10 mm (3.9 ± 1.9 mm), respectively, with a statistically significant difference. Examination for pathogens in the secretions from skin lesions showed that the three most common pathogens were *Candida albicans*, *Staphylococcus aureus*, and *Escherichia coli*. All patients recovered after antibiotic treatment.

**Conclusions:**

A blind cavity-like structure in the crypt may be related to excessive width of the preputial frenulum. Cryptitis may be a secondary infection caused by smegma trapped in the blind cavity-like structure. Maintaining cleanliness in the frenulum area may help to prevent the occurrence of cryptitis. Antibiotic treatment is effective.

## Résumé

**Contexte:**

Des maladies inflammatoires peuvent survenir dans la crypte située de chaque côté du frein du prépuce chez l’homme. Cette étude a été réalisée pour mieux comprendre l’étiologie, les manifestations cliniques et les méthodes de prise en charge de la cryptite localisée de chaque côté du frein préputial chez l’homme.

**Résultats:**

Treize patients traités pour une cryptite de chaque côté du frein du prépuce ont constitué le groupe d’observation, et 40 hommes en bonne santé le groupe témoin. La manifestation clinique des patients était la présence d’une substance huileuse jaunâtre incrustée dans la crypte. L’essuyage de la substance a révélé une structure conique en forme de cavité aveugle avec un diamètre d’ouverture de 1 à 5 mm (2,8±1,3 mm) et une profondeur de 1 à 4 mm (2,5±1,1 mm). Aucune structure aveugle ressemblant à une cavité n’a été trouvée dans les cryptes des hommes du groupe témoin. La plus courte distance entre les bords d’ouverture des cryptes bilatérales dans les groupes d’observation et contrôle était respectivement de 6 à 14 mm (10,3±2,4 mm) et de 2 à 10 mm (3,9±1,9 mm), avec une différence statistiquement significative. L’examen des agents pathogènes dans les sécrétions des lésions cutanées a montré que les trois agents pathogènes les plus courants étaient *Candida albicans*, *Staphylococcus aureus* et *Escherichia coli*. Tous les patients se sont rétablis après un traitement antibiotique.

**Conclusions:**

Une structure aveugle en forme de cavité dans la crypte peut être liée à une largeur excessive du frein préputial. La cryptite pourrait être une infection secondaire causée par le smegma piégé dans la structure aveugle en forme de cavité. Le maintien de la propreté dans la zone du frein du prépuce peut aider à prévenir l’apparition de la cryptite. Le traitement antibiotique est efficace.

## Introduction

The spatial structure enclosed by the preputial frenulum of the penis, the corona of the glans penis near the preputial frenulum, and the inner plate of the foreskin is a natural continuation of the coronal sulcus adjacent to the frenulum. This anatomical spatial structure located on either side of the preputial frenulum does not have a corresponding specialized term; we refer to it as the crypt beside the preputial frenulum. Approximately 15% of young males have pearly penile papules within the crypt [1] with the histopathological manifestation of angiofibroma [2]. This is a normal physiological phenomenon that occurs within the crypt. However, inflammatory diseases may also occur within the crypt. We conducted a study of male patients with cryptitis beside the preputial frenulum to understand the etiology, clinical manifestations, and management methods of cryptitis beside the preputial frenulum.

## Patients and methods

### Patients

The observation group comprised patients with cryptitis beside the preputial frenulum who were treated at the First People’s Hospital of Changshu from January 2010 to December 2022. The control group comprised healthy individuals who underwent routine health examinations during the same period.

The inclusion criteria were skin lesions within the crypt beside the preputial frenulum, with local redness, swelling, heat, and pain in the crypt. The exclusion criteria were phimosis, trauma, and infection by gonococcus, *Chlamydia trachomatis*, herpes virus, and *Treponema pallidum* as causes of cryptitis beside the preputial frenulum.

## Methods

### Clinical data

The following demographic data were recorded for both the observation group and control group: age, education, occupation, marital status, sexual exposure history, sexual orientation, sexual pattern, condom use, foreskin condition, and frequency of cleaning the glans.

**Fig. 1:**
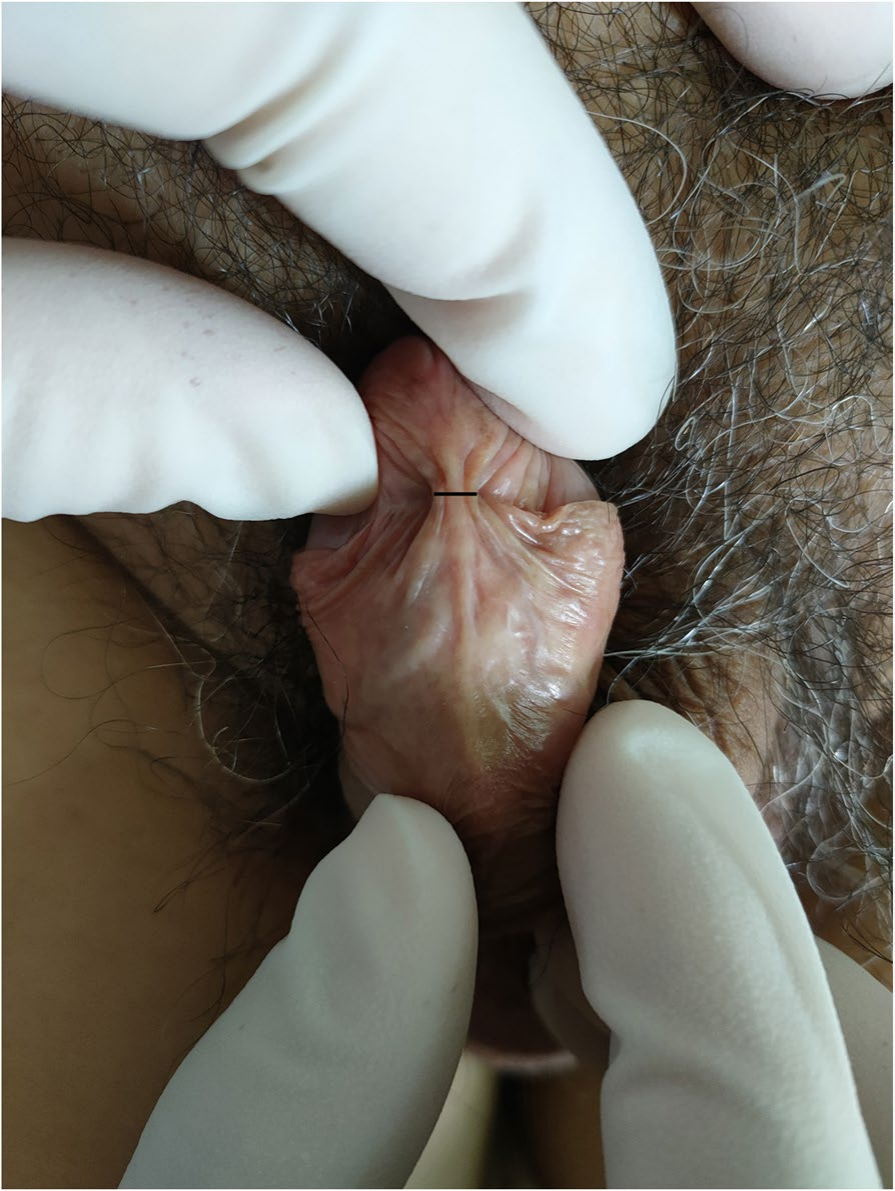
Measurement of the width of the preputial frenulum. The prepuce was retracted to expose the glans, preputial frenulum, and crypt beside the preputial frenulum. The shortest distance between the opening edges of the left and right crypts beside the preputial frenulum was measured with ruler as the width of the frenulum. The length of the black line is the width of the frenulum

The shortest distance between the opening edges of the left and right crypts beside the preputial frenulum was measured in both groups as follows. The participant was placed in the supine position to expose the external genitalia, and the penis was placed in dorsal extension. The prepuce was retracted to expose the glans, preputial frenulum, and crypt beside the preputial frenulum, and the shortest distance between the opening edges of the left and right crypts beside the preputial frenulum was measured as the width of the frenulum (Fig. 1).

The presence of a blind cavity-like structure at the crypt as well as the shape, diameter, and depth of the blind cavity-like structure were recorded and measured in both groups. Moreover, the occurrence site, manifestations of cryptitis beside the preputial frenulum, urinary pain, micturition frequency, urination urgency, and pyorrhea of the urethral orifice were recorded in the observation group.

### Laboratory examination

All specimens were subjected to Gram staining; microscopic examination was then performed to detect Gram-negative intracellular diplococci within phagocytes. All specimens were also cultured to detect general bacteria (excluding gonococcal species) and fungi. Genetic materials from gonococcal species, *Chlamydia trachomatis*, *Ureaplasma urealyticum*, *Mycoplasma hominis*, *M. genitalium*, and herpes simplex virus type 1 or 2 were detected using polymerase chain reaction. Dark-field microscopic examination was performed to detect *T. pallidum* in penile cutaneous lesion secretions from all patients. Venous blood samples were collected from all patients and subjected to analysis using a rapid plasma reagin test, *T. pallidum* hemagglutination assay [3], and human immunodeficiency virus antibody assay [4].

### Treatment

All patients’ lesions were washed with povidone-iodine. For fungal infections, the patients were prescribed oral itraconazole (0.2 g once a day for 7 days) along with external application of clotrimazole cream twice a day. For non-fungal infections, the patients were prescribed oral levofloxacin (0.5 g once a day for 7 days) along with external application of erythromycin eye ointment twice a day.

### Evaluation criteria for therapeutic effect

The patient was considered to have recovered when the skin lesions of the crypt had healed and the local redness, swelling, heat, and pain had disappeared. Treatment was considered to have failed if the skin lesions of the crypt had not healed or the local redness, swelling, heat, and pain in the area persisted.

### Statistical analysis

Data analysis was performed using R4.2.1 statistical software [5]. Rates were compared using chi square test. The normality of the shortest distance between the opening edges of the left and right crypts beside the preputial frenulum in both groups was tested using the Shapiro–Wilk method, and the rank sum test (Wilcoxon method) was used to compare the data that did not conform to a normal distribution. A *P* value of < 0.05 was considered statistically significant.

## Results

### Demographic and behavioral characteristics

The observation group comprised 13 patients aged 25 to 66 (34.0 ± 10.5) years. Their education levels were primary school (*n* = 3), middle school (*n* = 7), and high school (*n* = 3). All patients were married. All were heterosexual men who denied a history of sexual exposure and did not use condoms. The sexual pattern was genital–genital in all 13 patients. Their occupations were workers (*n* = 5), farmers (*n* = 4), and individual merchants (*n* = 4). Among them, nine (69.2%) patients had normal foreskin and four (30.8%) had a redundant prepuce; no patients had phimosis. The frequency of cleaning the glans and foreskin was once every 1 to 14 days (median, 3 days).

The control group comprised 40 patients aged 20 to 62 (36.6 ± 14.1) years, and all were married. Among them, 26 (65%) patients had normal foreskin and 14 (35%) had a redundant prepuce; no patients had phimosis. The frequency of cleaning the glans and foreskin was once every 1 to 5 days (median, 1 day).

There was no statistically significant difference in age (rank sum test: W = 279, *P* = 0.96) or the presence of a redundant prepuce (chi square = 0.08, *P* = 0.78) between the two groups. However, there was a statistically significant difference in the frequency of cleaning the glans and foreskin (rank sum test: W = 371.5, *P* < 0.01).

### Clinical characteristics

**Fig. 2:**
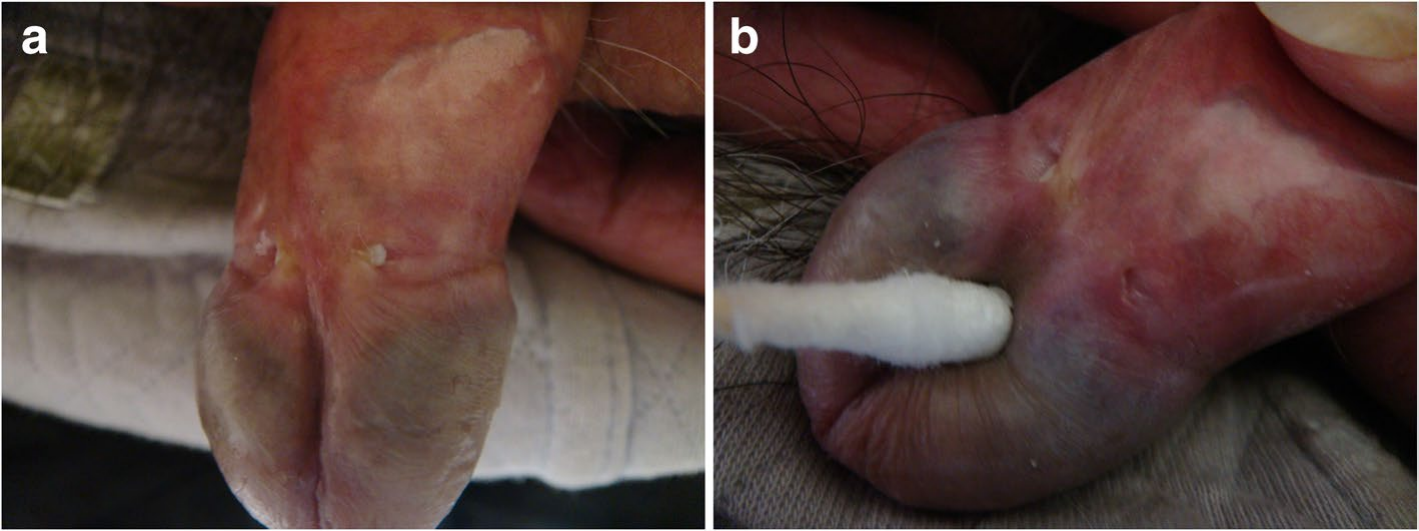
Manifestations of skin lesions in cryptitis. **A** Yellowish oily substance embedded in the bilateral crypts beside the preputial frenulum. **B** After removing the yellowish oily substance beside the preputial frenulum with a sterile cotton swab, a blind cavity-like structure was exposed, with local redness and swelling

In the observation group, the site of onset was the left crypt in five patients, the right crypt in six, and the bilateral crypts in two. In terms of clinical manifestations, all 13 patients had a yellow lipid substance embedded within the crypt beside the preputial frenulum (Fig. 2A). After wiping off the substance with a sterile cotton swab, a conical blind cavity-like structure was exposed (Fig. 2B). The blind cavity-like structure was not ruptured in 11 patients and was ruptured in 2 patients, and the structure was tender in all 13 patients. The blind cavity-like structure was located at the crypt apex in eight patients. All structures opened on the surface of the inner plate of the foreskin. The opening diameter was 1 to 5 mm (2.8 ± 1.3 mm), and the depth was 1 to 4 mm (2.5 ± 1.1 mm). Penile odor was present in all patients, but no patients had symptoms of urethritis. The shortest distance between the opening edges of the left and right crypts beside the preputial frenulum in the observation group was 6 to 14 mm (10.3 ± 2.4 mm), which was consistent with a normal distribution (W = 0.97, *P* = 0.86). The shortest distance between the opening edges of the left and right crypts beside the preputial frenulum in the control group was 2 to 10 mm (3.9 ± 1.9 mm), which was not consistent with a normal distribution (W = 0.87, *P* < 0.01). The difference between the two groups was statistically significant (rank sum test: W = 506.5, *P* < 0.01).

No blind cavity-like structure in the crypt beside the preputial frenulum was found in the control group.

### Results of laboratory examination

Specimens of the cutaneous lesion secretions were collected from all 13 patients in the observation group for Gram staining. No Gram-negative intracellular diplococci within phagocytes were observed in any of the cutaneous lesion secretion specimens. Polymerase chain reaction findings were negative for *Neisseria gonorrhoeae*, *Chlamydia trachomatis*, *U. urealyticum*, *M. hominis*, *M. genitalium*, and herpes simplex virus types 1 and 2. Dark-field microscopy did not reveal *T. pallidum* in any crypt cutaneous lesion secretions. The blood rapid plasma reagin test, *T. pallidum* hemagglutination assay, and human immunodeficiency virus antibody assay findings were negative in all patients. The results of bacterial and fungal cultures are shown in Table 1.

**Table 1:** Distribution and proportions of pathogens in 13 men with cryptitis beside the preputial frenulum

Pathogens	Cases	Proportion(%)
**Fungi**	3	23
*Candida albicans*	3	23
**Gram-positive bacteria**	5	39
*Staphylococcus aureus*	2	15
*Staphylococcus capitis*	1	8
*Staphylococcus haemolyticus*	1	8
*Streptococcus agalactiae*	1	8
**Gram-negative bacteria**	3	23
*Escherichia coli*	2	15
*Proteus mirabilis*	1	8
**No pathogen detected**	2	15

Examination for pathogens in the secretions from skin lesions showed that the three most common pathogens were *Candida albicans*, *Staphylococcus aureus*, and *Escherichia coli*

### Treatment results

All 13 patients were cured after treatment. All three patients with *Candida albicans* infections recovered 7 days after treatment. All 10 patients with non-fungal infections recovered within 4 to 10 (6.70 ± 2.21) days after treatment.

## Discussion

The preputial frenulum has no definite anatomical starting or ending point and manifests as a cord-like longitudinal skin fold of unequal width. Although its length and width cannot be measured, the shortest distance between the openings of the bilateral crypts beside the preputial frenulum can be measured as the width of the preputial frenulum. Notably, the preputial frenulum should be in a tension state that avoids local deformation of the glans or urethral opening during this measurement. In this study, the preputial frenulum was wider in the observation group than in the control group, and the wider frenulum covered more of the inner plate of the foreskin next to the preputial frenulum to form a blind cavity-like structure at the crypt. Whether the formation of this blind cavity-like structure is related to the short preputial frenulum and the deviation of the attachment point of the preputial frenulum to the side of the penis midline requires further study. The formation of blind cavity-like structures may be primary and related to ontogeny. However, when smegma becomes embedded in the crypt beside the preputial frenulum, the fascial tissue at the top of the crypt may adhere too tightly to the tunica albuginea of the urethral cavernous body, resulting in secondary formation of a blind cavity-like structure.

Tyson’s glands are a pair of hairless sebaceous glands located on either side of the preputial frenulum [6]. Gonococcal tysonitis is a local complication of urethral gonorrhea [7]. The function of Tyson’s glands is to produce smegma [8]. Smegma is a normal secretion that is mainly composed of exfoliated epithelial cells, fat, and protein. According to the relevant literature [9–11], smegma is harmless and non-irritating, and it can even help protect and lubricate the glans and inner foreskin and promote erection, inversion, and penetration during sexual intercourse. However, the yellowish oily substance constantly secreted by these glands becomes mixed with epithelial cells that have shed from the foreskin and residual urine. Over time, this material accumulates, thickens, and tightly adheres to the inner plate of the foreskin and glans. Smegma can become colonized by a variety of mixed bacteria, 50% of which are smegma bacilli [9]. The smegma in children is also colonized by various uropathogens, among which *Escherichia coli* is the main pathogen [12]. Research has shown that smegma is an accumulation of cellular debris in the preputial fold and has a dual role in preputial stone formation [13]. In addition to functioning as a nidus, smegma can be a direct irritant that induces inflammation, adhesions, and preputial stenosis, leading to obstruction with stasis [14]. Therefore, if the smegma in the blind cavity-like structure in the crypt is not removed in a timely manner, it may cause secondary infection of the crypt and even crypt stones [15, 16]. The cryptitis occurs next to the preputial frenulum; thus, it belongs to the category of balanoposthitis. Balanoposthitis has a wide variety of etiologies, including both infectious and noninfectious conditions [17]. An uncircumcised state is considered a major predisposing factor for balanoposthitis, and poor hygiene, buildup of smegma, and a tight foreskin favor this inflammatory condition [18]. Preputial stone disease is the rarest type of urolithiasis. Men with severe phimosis and poor hygiene are mainly affected. Symeonidis et al. [19] emphasized the importance of personal hygiene in the prevention of preputial stone formation. *Candida* species are the most commonly isolated microorganisms in patients with balanoposthitis [18]. In previous studies, decreased hydration of the mucosa of the glans and an increased pH were found in patients with balanoposthitis. Additionally, the balanoposthitis was associated with impairment of the physical barrier provided by the male genital mucosa and a higher colonization rate of *Staphylococcus warneri* and *Prevotella bivia* [20]. Therefore, a possible treatment regimen for balanoposthitis may include restoration of both the genital mucosa barrier and the balance of the indigenous microbial population [20]. Because of limitations of our study conditions, we did not measure the mucosal pH, transepidermal water loss, or mucosal hydration in the crypts adjacent to the preputial frenulum. We detected a total of 7 pathogens in 13 patients. The three most common pathogens were *Candida albicans* (23%), *S. aureus* (15%), and *E. coli* (15%). *Candida albicans* and *S. aureus* are dangerous pathogens that are responsible for a variety of infections. Accordingly, *Candida albicans* and *S. aureus* isolated from the discharge of the inflammatory lesions within the crypts may be causative pathogens of this disease. The other five pathogens detected in this study could not be judged as pathogenic bacteria, contaminating bacteria, or normal resident bacteria.

In this study, the 13 patients in the intervention group, all of whom had a low education level, cleaned their glans and foreskin once every 1 to 14 days (median, 3 days), whereas the patients in the control group cleaned their glans and foreskin once every 1 to 5 days (median, 1 day); there was significant difference in the cleaning frequency between the two groups. Failure to pay attention to the cleanliness and hygiene of the external genitalia may be one cause of inflammation of the crypts. Therefore, health education is necessary. For patients who have deep and narrow blind cavity-like structures, repeated recurrence of cryptitis, and lack of clinical resolution after cleaning and drug treatment, surgical resection of excessively deep crypts or penile frenuloplasty can be considered to achieve a change in the shape of the crypts. The effects of these surgical treatments need to be examined in further clinical studies.

The main limitations of this study are the small number of cases and the lack of histopathological examination because of the anatomical location of the lesions.

## Conclusion

A blind cavity-like structure in the crypt may be related to excessive width of the preputial frenulum. Cryptitis may be a secondary infection caused by smegma trapped in the blind cavity-like structure. Maintaining cleanliness in the frenulum area may help to prevent the occurrence of cryptitis. Antibiotic treatment is effective.

## Data Availability

The study’s data is available upon request.
